# Thickness-dependent transport channels in topological insulator Bi_2_Se_3_ thin films grown by magnetron sputtering

**DOI:** 10.1038/srep25291

**Published:** 2016-05-04

**Authors:** Wen Jie Wang, Kuang Hong Gao, Zhi Qing Li

**Affiliations:** 1 Tianjin Key Laboratory of Low Dimensional Materials Physics and Preparing Technology, Department of Physics, Tianjin University, Tianjin 300072, China

## Abstract

We study the low-temperature transport properties of Bi_2_Se_3_ thin films grown by magnetron sputtering. A positive magnetoresistance resulting from the weak antilocalization (WAL) effect is observed at low temperatures. The observed WAL effect is two dimensional in nature. Applying the Hikami-Larkin-Nagaoka theory, we have obtained the dephasing length. It is found that the temperature dependence of the dephasing length cannot be described only by the Nyquist electron-electron dephasing, in conflict with prevailing experimental results. From the WAL effect, we extract the number of the transport channels, which is found to increase with increasing the thickness of the films, reflecting the thickness-dependent coupling between the top and bottom surface states in topological insulator. On the other hand, the electron-electron interaction (EEI) effect is observed in temperature-dependent conductivity. From the EEI effect, we also extract the number of the transport channel, which shows similar thickness dependence with that obtained from the analysis of the WAL effect. The EEI effect, therefore, can be used to analyze the coupling effect between the top and bottom surface states in topological insulator like the WAL effect.

Prototypical three-dimensional topological insulators (TIs) such as Bi_2_Se_3_ and Bi_2_Te_3_^1^,^2^ are characterized by gapped bulk states and gapless surface states, which have been established by angle resolved photoemission spectroscopy^3^,^4^,^5^. The gapless surface state is topologically protected by the time reversal symmetry. Regarding the transport property of TIs, the time reversal symmetry can be suppressed in small perpendicular magnetic fields, giving rise to a positive magnetoresistance (MR). This is the well-known weak antilocalization (WAL) effect^6^,^7^,^8^,^9^,^10^. Because of intrinsic defects or unintentional doping, the bulk states are usually conductive, leading to the coupling effect between the top and the bottom surface states beyond hundreds of nanometer^11^. Therefore, a multi-channel model of the Hikami-Larkin-Nagaoka (HLN) theory is usually adopted to analyze the MR to extract the number of the transport channels^12^,^13^,^14^,^15^,^16^. This is critical to determine the coupling strength between the top and the bottom surface states^11^,^17^,^18^,^19^. On the other hand, the electron-electron interaction (EEI) effect has recently been observed in topological insulator thin films^20^,^21^,^22^,^23^. From the EEI effect, one can also extract the number of the transport channels^21^. However, a comparison between the results of WAL and EEI effects is rarely carried out.

Topological insulator Bi_2_Se_3_ has been studied extensively due to its the large bulk bandgap of 0.3 eV^24^,^25^,^26^. Experimentally, Bi_2_Se_3_ has been synthesized by both the molecular beam epitaxy (MBE)^27^ and the Bridgman method^5^,^8^,^28^. However, both techniques are not suitable to prepare the large-area thin films of TIs for the industrial applications. For the production of the large-area thin films, the magnetron sputtering has been widely used owing to its low cost and relatively simple process. However, there has no report on the properties of topological insulator Bi_2_Se_3_ prepared by magnetron sputtering method until now.

In this paper, topological insulator Bi_2_Se_3_ thin films have been grown on SrTiO_3_(111) substrate by the rf-magnetron sputtering method. At low temperatures, the two dimensional (2D) WAL effect has been observed. Applying the HLN theory, we have extracted the dephasing length, the temperature dependence of which cannot be described only by the Nyquist electron-electron dephasing mechanism. Meanwhile, the numbers of the transport channels are extracted. It is found the numbers of the transport channels increase with increasing the thickness of the films, which is consistent with that obtained from the analysis of the EEI effect.

## Results and Discussion

**Figure 1:**
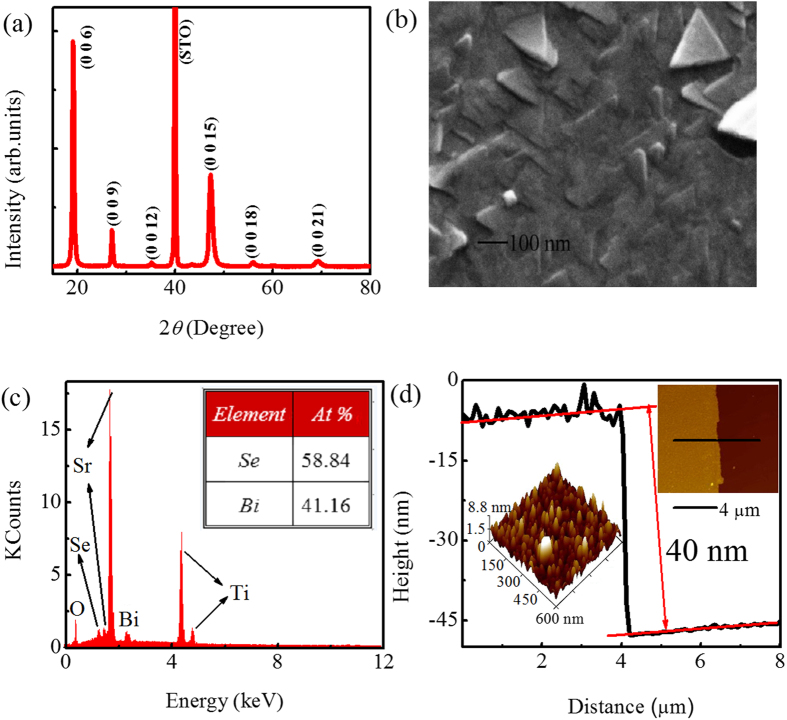
(**a**) XRD pattern, (**b**) SEM image, and (**c**) EDS of a representative Bi_2_Se_3_ thin film with the *t* = 36 nm grown on a SrTiO_3_(111) substrate. (**d**) AFM topography with the height profile across a 40 nm thick Bi_2_Se_3_ thin film. The left and right insets are the 3D and 2D topographies, respectively.

Figure 1(a) shows the x-ray diffraction (XRD) pattern of a representative Bi_2_Se_3_ thin film with thickness *t* = 36 nm. The diffraction peaks of (0, 0, 3*n*) indicates the rhombohedral structure and the thin film growth along the [001] direction. The sharp XRD peaks manifest the high crystal quality of our films. Figure 1(b) reveals the scanning electron microscopy (SEM) image of the thin film. The surface of the film is composed of triangular domains, reflecting the three-fold symmetry of the film, the same as reported works^29^,^30^. The energy dispersive x-ray spectroscopy (EDS) is shown in Fig. 1(c), where Se and Bi peaks are observed. From these peaks, the ratio of Se to Bi is found to be 1.43 that is near to 1.5 for the stoichiometric Bi_2_Se_3_. However, it should be mentioned that the peaks of both Sr and Ti originating from the SrTiO_3_ substrate are high compared with Se and Bi peaks since the Bi_2_Se_3_ sample is thinner (36 nm). Figure 1(d) shows one representative atomic force microscopy (AFM) topography with height profile across the ~40 nm thick Bi_2_Se_3_ thin film. The root mean square roughness of the surface is ~2.24 nm, which is obtained from the 3D topography of the AFM measurement shown in the left inset of Fig. 1(d). It can be concluded that, by using magnetron sputtering method, we obtain Bi_2_Se_3_ samples with the high crystal quality, comparable to the MBE-grown Bi_2_Se_3_ films.

**Figure 2:**
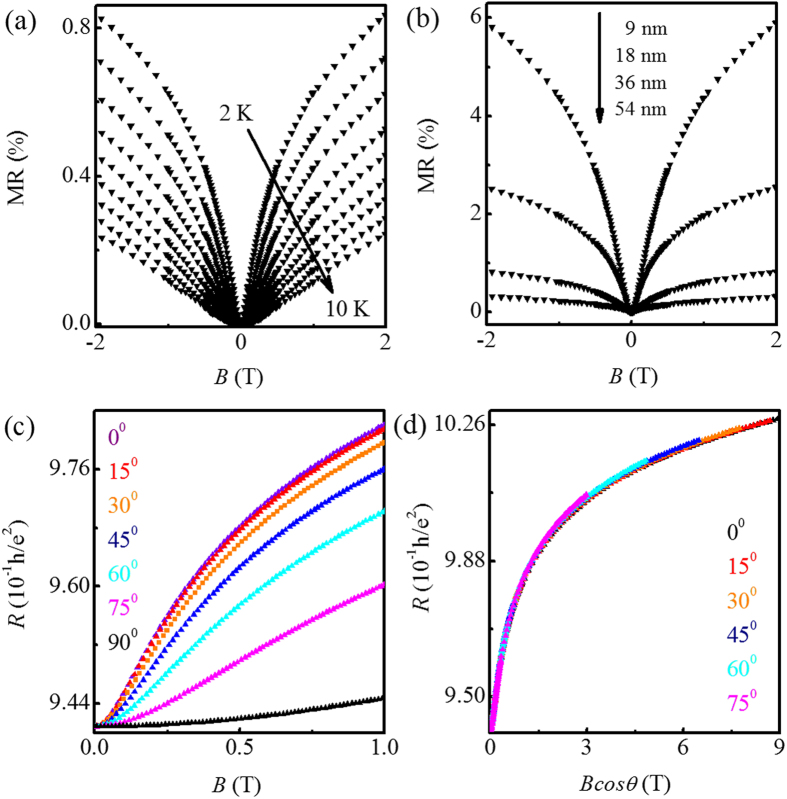
(**a**) The MR of a representative Bi_2_Se_3_ thin film with the *t* = 36 nm in low-field range at various temperatures. (**b**) Variation of the MR at 2 K for different thicknesses in low-field range. (**c**) The MR at 2 K for various tilted fields. (**d**) The MR as a function of the perpendicular field component at 2 K for various tilted angles (0°–75°).

Figure 2(a) shows the magnetoresistance MR [*MR* = [*R*(*B*) − *R*(0)]/*R*(0) × 100] of a representative sample with *t* = 36 nm at various temperatures. In the low field regime, a sharp increase in the MR appears with increasing magnetic field at 2 K. And the increase is gradually suppressed with increasing temperature, which is a characteristic of the WAL effect^30^,^31^. Figure 2(b) plots the MR of four samples with different thicknesses at 2 K in a perpendicular magnetic field. It can be seen that all MR curves show the positive MR of the WAL effect in the low field range. But when the thin film becomes thicker, the magnitude of MR remarkably decreases, which is similar to the observations in the films grown by the MBE^22^. The observed MR of the WAL effect was studied in tilted magnetic fields. Figure 2(c) shows MR of a representative sample at 2 K for various tilted fields. From the figure, one can see that the positive MR is gradually suppressed with increasing *θ* from *θ* = 0° (magnetic field is perpendicular to the plane). For *θ* = 90° (magnetic field is in plane), the WAL cusp in MR completely disappears. Figure 2(d) exhibits MR as a function of the perpendicular component of magnetic field at different tilted fields. It can be seen that all the MR curves coincide with each other. This clearly manifests that the observed WAL effect is 2D in nature^32^,^33^.

**Figure 3:**
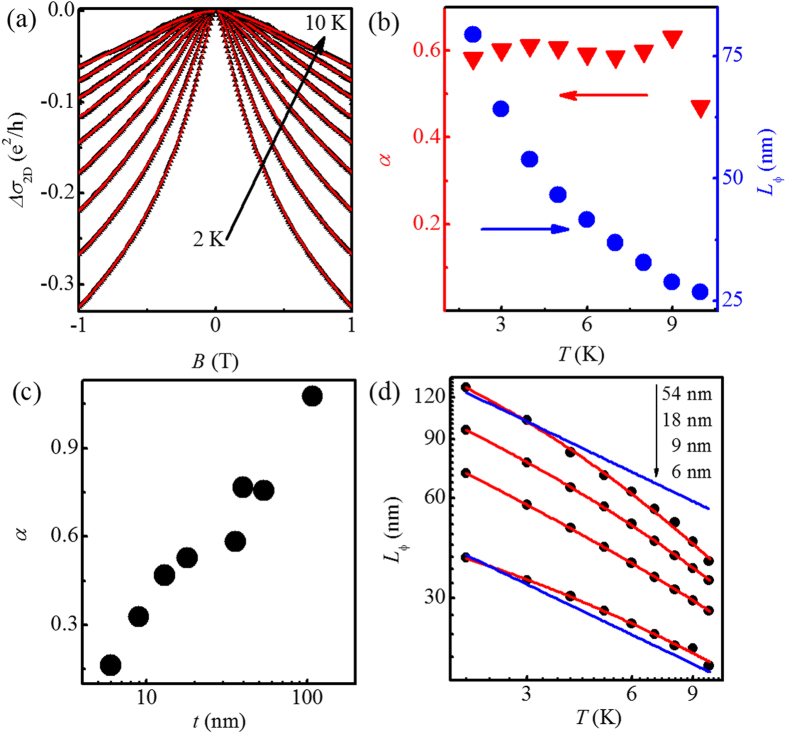
(**a**) The magnetoconductivity of a representative Bi_2_Se_3_ thin film with the *t* = 36 nm in low-field range at various temperatures. The red solid lines are the theoretical fits. (**b**) The extracted parameters *α* and *L*_*ϕ*_ versus temperature. (**c**) The extracted *α* as a function of *t* at 2 K. (**d**) 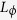 as a function of temperature for Bi_2_Se_3_ thin films with different thicknesses. The red solid lines are the fits by using Eq. (2), and the blue solid lines are the fits with the relation *L*_*ϕ*_ ∝ *T*^−1/2^.

For a 2D system, the WAL effect can be fitted to the standard HLN theory^6^:


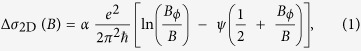


where α is a coefficient, 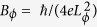, *e* is the electronic charge, 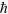 is the reduced Planks constant, *L*_*ϕ*_ = (*Dτ*_*ϕ*_)^1/2^ is the phase coherence length (here, *D* and *τ*_*ϕ*_ are the electron diffusion constant and the electron dephasing time, respectively), and *ψ*(*x*) is the digamma function. Equation (1) has been widely used to analyze the WAL effect in 3D TIs. It is worthy to note that Adroguer *et al.*^34^ recently calculated the WAL effect of the TIs for a single surface state in the presence of spin-orbit impurities and obtained new formula that is different from the HLN theory. However, the electron density determined from the Hall resistance is in the order of 10^15^ cm^−2^ for all our samples, which indicates the Fermi level is located in the conduction band^35^ and thus bulk states cannot be negligible. This, combined with the usual existence of two surface states (i.e., top and bottom surface sates) for topological insulator, makes the theory of Adroguer *et al.* invalid to analyze our data. Furthermore, we calculate the mean free path for all our samples and find that the maximum is 23.8 nm, which is far smaller than the distance (3.6 mm) between the positive and negative voltage contacts of the thin films. This indicates that the transport is in the diffusive regime. Meanwhile, the values of *k*_*F*_*l* for all our samples varying between 2.4 and 413.9 (>1) indicate that the transport is in weakly disordered regime. Therefore, we use Eq. (1) to study the WAL effect here. The value of *α* in Eq. (1) should is equal to 0.5 for a single coherent transport channel, and 1 for two independent coherent transport channels^32^. In TI thin film, two gapless surface states can be regarded as two transport channels. Thus *α* = 1 should be obtained from the fit using Eq. (1). In practice, however, the obtained values of *α* are usually smaller than unity because of the coupling effect between the top and bottom surfaces^35^,^36^. In order to analyze our MR data by using Eq. (1), we obtain the magnetoconductivity by 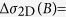

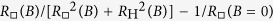
, where *R*_◻_ and *R*_H_ are respectively the sheet resistance and the Hall resistance. It can be seen that the magnetoconductivity of the representative sample with *t* = 36 nm in Fig. 3(a) can be well fitted to Eq. (1) (red solid lines are fit curves) at various temperatures from 2 up to 10 K, above which we cannot obtain a reliable fit due to the weaker WAL effect. From the fits, we extracted *α* and *L*_*ϕ*_. As shown in Fig. 3(b), the extracted *α* (triangles) is found to be ~0.6, independent of temperature.

Figure 3(c) exhibits the extracted *α* varying with *t* of the thin films at 2 K. For all our samples, the relation *L*_*ϕ*_ > *t* is obtained at 2 K (e.g., *L*_*ϕ*_ = 159 nm for the thickest film of *t* = 108 nm), which is suggestive of 2D coherent process. The extracted *α*, therefore, is reliable by using Eq. (1). As seen in the figure, *α* monotonically increases from 0.16 to 1.08 with increasing thickness from *t* = 6 to 108 nm. For the thickest sample (i.e., *t* = 108 nm), *α* = 1.08 is near to unity, corresponding to two channels. This indicates that the top and bottom surface states can be regarded as two separate channels and no coupling occurs between them. On decreasing *t* from 108 to 13 nm, *α* continually decreases from 1.08 to 0.5, suggesting that two channels are converged into one channel. This is likely to result from the gradually enhanced intersurface coupling on decreasing *t*, as has been reported in Cu-doped Bi_2_Se_3_ samples^11^. Since the direct coupling between two surfaces usually occurs at *t* < 10 nm, the intersurface coupling in our thin films must be mediated by the bulk states^22^, which is different from the direct interlayer tunneling. That is, an indirect coupling occurs for *t* ≥ 13 nm, and similar results are reported recently in Bi_2_Te_2_Se nanoribbons^17^. When *t* = 13 nm, *α* = 0.5 is obtained, implying that the indirect coupling are so strong that these two surface states act as a single transport channel. On further decreasing *t* lower to 13 nm, *α* should manifest a saturation with *α* = 0.5 because the direct intersurface coupling may occur^35^. When *t* < 13 nm as shown in Fig. 3(c), however, *α* is found to be smaller than 0.5, which has been widely reported in Bi_2_Se_3_ and Bi_2_Te_3_ materials^9^,^22^,^30^,^31^,^32^,^33^,^36^,^37^,^38^. This may be induced by (1) the gap opening of the surface state at the Dirac point^27^,^37^,^38^ or (2) the enhanced disorder in the thinner film^39^,^40^. According to Zhang *et al.*^41^, the gap opening usually occurs when *t* < 5 nm. Considering that *t* ≥ 6 nm for all our samples, the latter (i.e., the enhanced disorder) is likely to cause the smaller *α*(<0.5) in our samples.

The extracted *L*_*ϕ*_ (circles) from the fits is shown in Fig. 3(b), which is expected to decrease with increasing temperature due to increased inelastic scattering^13^,^42^. Theoretically, the Nyquist electron-electron dephasing dominates in 2D system, and performs as *L*_*ϕ*_ ∝ *T*^−1/2^(i.e., *τ*_*ϕ*_ ∝ *T*^−1^)^43^,^44^. Experimentally, the relation *L*_*ϕ*_ ∝ *T*^−1/2^ has been widely reported in topological insulators. In conflict with the prevailing experimental results, however, we unexpectedly find that *L*_*ϕ*_ exhibits a deviation from this relation, with the smaller value in high temperature range as shown in Fig. 3(d) for the film with *t* = 54 nm (the blue solid line is a fit with *L*_*ϕ*_ ∝ *T*^−1/2^). For this deviation, there are three other possible scattering components in the electron dephasing process: (1) the large-energy-transfer electron-electron scattering, (2) 3D electron-phonon scattering and (3) the 2D electron-phonon scattering. For large-energy-transfer electron-electron scattering, the relation *τ*_*ϕ*_ ∝ *T*^−2^ can be obtained when *k*_*B*_*Tτ*_*e*_/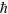 >1 (here, *τ*_e_ is the momentum relaxation time)^43^. Namely, the power laws *L*_*ϕ*_ ∝ *T*^−*x*/2^ with *x* = 2 can be obtained when *k*_*B*_*Tτ*_*e*_/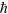
> 1. Here, the *x* > 1 indicates that one can obtain the smaller theoretical value of *L*_*ϕ*_ in our studied temperature range, which might suppress the deviation as shown in Fig. 3(d). To clarify whether there exists large-energy-transfer component in the dephasing process, we calculate the value for *k*_*B*_*Tτ*_*e*_/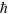 and find that it less than 10^−4^ (i.e., *k*_*B*_*Tτ*_*e*_/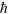
< 1) for all our samples in the temperature range of 2–10 K. However, this cannot ensure that there is no large-energy-transfer component in each transport channel because *τ*_e_ is obtained from the Hall mobility that includes the contribution from all the three channels (i.e., the bulk state, the top and bottom surface states). Particularly, high mobility in surface states can induce the larger value for *k*_*B*_*Tτ*_*e*_/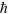, which thus may give rise to the large-energy-transfer scattering. However, the reported mobility of Bi_2_Se_3_ surface states is small, which varies between 13 and 3000 cm^2^v^−1^s^−1^ 11,22,27,37,44. From these reported values of the mobility, we calculate the lowest temperature at which the large-energy-transfer scattering starts and find that it is larger than 30 K, which is beyond our studied temperature range of 2–10 K. Therefore, the large-energy-transfer scattering may not play dominant part in the dephasing process.

It should be noted that the magnitude of the extracted *L*_*ϕ*_ is slightly smaller than *t* near 10 K for four thicker samples (i.e., thin films with *t* = 36, 40, 54, and 108 nm). For example, for the thin film with *t* = 54 nm as shown in Fig. 3(d), *L*_*ϕ*_ varies between 50 and 39 nm in the temperature range of 8–10 K. This indicates a smooth crossover from 2D to 3D WAL effect on increasing temperature, as has been observed in GaAs thin film^45^. Therefore, there may be the 3D electron-phonon scattering component in the dephasing process, while the electron-electron scattering is usually negligible in 3D system^46^. For the 3D electron-phonon scattering, the power law *L*_*ϕ*_ ∝ *T*^−*x*/2^ with *x* = 3 (i.e.,*τ*_*ϕ*_ ∝ *T*^−3^) should be obtained^47^ due to the excited longitudinal phonon^48^. That the index *x* = 3 can induce the smaller theoretical value of *L*_*ϕ*_, which may explain the observed deviation in Fig. 3(d). Thus, the 3D electron-phonon scattering may have contribution to dephasing process in our samples.

For the electron-phonon scattering in a 2D system, the power laws *L*_*ϕ*_ ∝ *T*^−*x*/2^ with *x* > 1 have also been proposed for the electron-phonon dephasing in some theories^47^,^49^. Experimentally, the index *x* ~2–3 has been observed in the phase-change material GeSb_2_Te_4_^50^. That the index *x* ~2–3 can induce the smaller theoretical value of *L*_*ϕ*_, too. The 2D electron-phonon scattering, therefore, may play a role in the dephasing process^16^. On the other hand, when the film becomes thinner, *L*_*ϕ*_ shows a weaker temperature dependent. As shown in Fig. 3(d) for the films with different *t*, the increase in *L*_*ϕ*_ with decreasing temperature is gradually suppressed with decreasing *t*. For the thinnest film (i.e., *t* = 6 nm), *L*_*ϕ*_ as a function of *T* also deviates from the relation *L*_*ϕ*_ ∝ *T*^−1/2^, with the slightly larger value in high temperature range. This suggests that there must be a contribution of temperature-independent dephasing term^43^,^50^, which has been observed recently in vapor phase deposited Bi_2_Se_3_^51^. It can be concluded that, in additional to the aforementioned Nyquist electron-electron dephasing, the 3D electron-phonon scattering, the 2D electron-phonon scattering, and the temperature-independent dephasing term may exist in our samples. Therefore, the extracted *L*_*ϕ*_ with different *t* can be fitted to the following equation^50^:


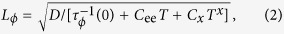


where 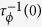 is the temperature-independent term, *C*_ee_*T* is the electron-electron dephasing term, and *C*_*x*_*T*^*x*^ is the 3D electron-phonon and/or the 2D electron-phonon dephasing term. As seen in Fig. 3(d), our experimental data can be well described by Eq. (2) (the red solid lines are the fits). And the fitted values of 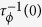, *C*_ee,_ *C*_*x*,_ and *x* are shown in Table 1. The index *x* varies between 2 and 3, consistent with the observations in GeSb_2_Te_4_^50^. This indicates that there exists the 2D electron-phonon scattering component in the dephasing process, while the 3D electron-phonon scattering component is negligible.

**Figure 4:**
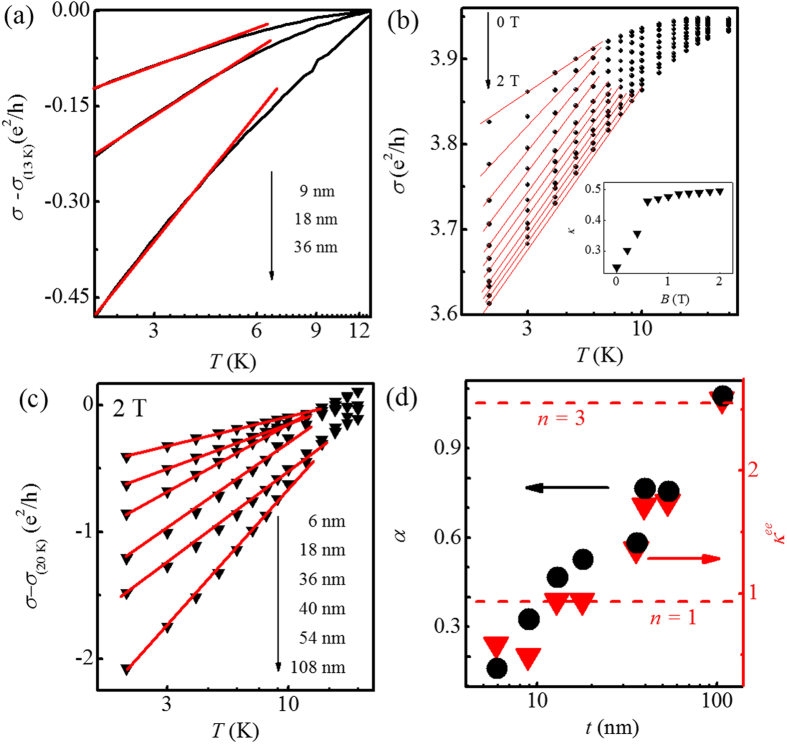
(**a**) Measured logarithmic temperature-dependent conductivity for Bi_2_Se_3_ thin films at low temperature. The red solid lines are guides for the eye. (**b**) Measured logarithmic temperature-dependent conductivity of a representative thin film with the *t* = 9 nm at perpendicular magnetic field which increases from 0 to 2 T by a step of 0.2 T. The solid lines are a guide for the eye. Inset: slope *κ* = (*πh*/*e*^2^)(∂*σ*/∂ln *T*) obtained by linear fitting the curves in main panel of (**b**) plotted as a function of magnetic field. (**c**) Variation of 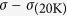 at 2 T with temperature for different thicknesses. (**d**) Comparison between the extracted *α* and *κ*^*ee*^ as a function of *t*. Two red dashed horizontals mark two *κ*^*ee*^ corresponding to *n* = 1 and *n* = 3, respectively.

Meanwhile, the WAL effect-dominated conductivity should increase with decreasing temperature without external magnetic field^52^. As shown in Fig. 4(a), however, the conductivity demonstrates a logarithmic decrease as temperature is lowered. This indicates that the EEI effect dominates the temperature dependence of the conductivity, which has been observed in topological insulators such as Bi_2_Te_3_^36^,^42^, Bi_2_Se_3_^20^,^22^,^23^,^35^, and Sb_2_Te_3_^21^. Although the relative permittivity of Bi_2_Se_3_ single crystal is found to be high^53^, the Bi_2_Se_3_ thin film cannot be insulating without doping due to Se vacancy. As mentioned above, the high electron density in the order of 10^15^ cm^−2^ in our thin films is obtained. Therefore, it is understandable to observe the EEI effect here. In addition, we calculate the thermal diffusion length *L*_*T*_ (
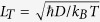
) in the temperature range of 2–10 K and find that *L*_*T*_ > *t* for all our samples. For example, the value of *L*_*T*_ for the thickest sample varies between 362 and 808 nm in the temperature range of 2–10 K, which is far larger than the corresponding film thickness of *t* = 108 nm. This indicates that the observed EEI effect is 2D in nature. Theoretically, the 2D EEI correction to the conductivity is given by^21^,^46^


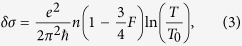


where *n* is the number of the transport channels for the EEI effect, *F* is the electron screening factor (0 < *F* < 1), and *T*_0_ is the characteristic temperature. Figure 4(b) shows the logarithmic temperature dependence of the conductivity for a representative sample with *t* = 9 nm at various magnetic fields. On increasing magnetic field from 0 to 2 T, the slope of the *σ*(*T*) curve firstly becomes enhanced rapidly and then tends to saturate. In order to describe this change quantitatively, the slope can be defined by *κ* = (*πh*/*e*^2^)(∂*σ*/∂ln *T*). The *κ* obtained from the linear fits (red solid lines) in Fig. 4(b) are plotted in the inset of Fig. 4(b) as a function of magnetic field. A sharp increase at low magnetic fields can be attributed to the rapid suppression of the WAL effect. And the saturated value of *κ* in high field range indicates that the WAL is quenched^42^. Then the saturated *κ*, referred to *κ*^*ee*^, only includes the EEI correction to the conductivity at high field. Consequently, *κ*^*ee*^ = *n*(1 − 3*F*/4), according to Eq. (3).

Figure 4(c) shows the logarithmic temperature-dependent normalized 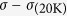 at magnetic field *B* = 2 T for the thin films with different film thicknesses. As seen in the figure, there is an apparent increase in *κ*^*ee*^ with increasing *t*. The *t* dependent magnitude of *κ*^*ee*^ is given in Fig. 4(d) (triangles). Clearly, *κ*^*ee*^ roughly increases from 0.49 to 2.58 with increasing *t* from 6 to 108 nm. According to the relation *κ*^*ee*^ = *n*(1 − 3*F*/4), the maximum (i.e., 2.58) of *κ*^*ee*^ requires *n* > 2 to assure *F* > 0. Then assuming *n* = 3, corresponding to three transport channels for the EEI effect, we obtain *F* = 0.19, which is comparable to the reported values of 0.15 (ref. 23) and 0.27 (ref. 35). Therefore, when *t* = 108 nm, there must be three independent transport channels for the EEI effect, including the top and bottom surface states and the bulk state. As *t* is reduced, *κ*^*ee*^ exhibits a decrease, which is suggestive of a decrease in the number of the transport channels for the EEI effect.

Particularly, it can be distinctly seen in Fig. 4(d) that *κ*^*ee*^ (triangles) has the similar *t* dependence with *α* (circles). This demonstrates the close relation between the WAL effect and the EEI effect. For the thin film with *t* = 108 nm, the top and bottom surface states and the bulk state can be regarded as three independent EEI transport channels, corresponding to *κ*^*ee*^ = 2.58 (equivalently, *n* = 3). Meanwhile, there are only two WAL transport channels (corresponding to *α* = 1) as has been discussed above since the bulk state has no contribution to the WAL effect^11^,^15^ and there is no coupling between the top and bottom surface states. As *t decreases* from 108 to 13 nm, the indirect coupling between the top and bottom surface states through bulk states occurs, which not only influences the WAL effect as discussed above but also influences the EEI effect. As seen in Fig. 4(d), *κ*^*ee*^ (triangles) gradually decreases from 2.58 (corresponding to *n* = 3) to 0.86 (corresponding to *n* = 1) on decreasing *t* from 108 to 13 nm. This also indicates that the strength of indirect coupling between the top and bottom surface states are enhanced with decreasing the thickness of the samples, which effectively reduce the number of the EEI transport channels. When *t* < 13 nm, direct coupling between the top and bottom surface states should occur expectedly, which makes the whole system as a single EEI channel, as well as a single WAL channel as discussed above. However, *κ*^*ee*^ < 0.86 (corresponding to *n* < 1) is obtained as seen in Fig. 4(d) when *t* < 13 nm, which might be related to the stronger disorder in thinner films like the WAL effect and further study is needed.

## Conclusions

In conclusion, Bi_2_Se_3_ thin films with different thicknesses were prepared on SrTiO_3_(111) substrate by the rf-magnetron sputtering. The 2D WAL effect is observed at low temperature. Applying the HLN theory, we extracted *L*_*ϕ*_ and *α*. From the extracted *L*_*ϕ*_ as a function of temperature, we found that, in additional to the Nyquist electron-electron scattering, the 2D electron-phonon scattering also contributes to the electron dephasing in our samples. The extracted *α* increases with increasing *t*, exhibiting the thickness-dependent number of the transport channels due to coupling between the top and bottom surface states. Meanwhile, the EEI effect is observed at low temperature. From this effect, we also extracted the number of the transport channels, which shows an increase with increasing *t* again. That is, the WAL and EEI effects consistently exhibit the transport channel number with varying the thickness of Bi_2_Se_3_ films.

## Methods

Bi_2_Se_3_ thin films were deposited on SrTiO_3_(111) substrate by the rf-magnetron sputtering. A commercial Bi_2_Se_3_ target (99.99% purity) was used as the sputtering source. The base pressure of the vacuum chamber was ≤9 × 10^−5^ Pa, and the sputtering deposition was carried out in an argon atmosphere (99.999%) of 0.3 Pa. During sputtering, the substrate temperature was kept at 425 °C. Hall-bar-shaped samples were defined using mechanical masks. For the thickness-dependent research, the thin films with thickness varying between 6 and 108 nm were deposited at an average growth rate of ~3 nm/min. The thicknesses of the thin films were measured by the AFM. The structure, composition, and surface morphologies of the thin films were characterized by the XRD, the EDS, and the SEM. The four-probe electrical conductivity and Hall effect measurements were carried out by using a physical property measurement system (PPMS-6000, Quantum Design).

## Additional Information

**How to cite this article**: Wang, W. J. *et al.* Thickness-dependent transport channels in topological insulator Bi_2_Se_3_ thin films grown by magnetron sputtering. *Sci. Rep.* **6**, 25291; doi: 10.1038/srep25291 (2016).

## Figures and Tables

**Table 1:** The fitting parameters 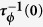 , *C*_ee_, *C*_*x*_, and *x*.

**Thickness (nm)**	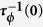 **(s**^**−1**^)	***C***_**ee**_ **(K**^**−1**^ **s**^**−1**^)	***C***_***x***_ **(K**^**−*****x***^ **s**^**−1**^)	***x***
_6_	1.65 × 10^11^	5.27 × 10^11^	1.02 × 10^10^	2.06
_9_	2.18 × 10^10^	3.24 × 10^11^	9.07 × 10^9^	2.18
_13_	3.54 × 10^11^	1.15 × 10^12^	4.11 × 10^10^	2.24
_18_	1.17 × 10^11^	6.66 × 10^11^	3.00 × 10^10^	2.27
_36_	1.34 × 10^11^	8.49 × 10^11^	4.36 × 10^10^	2.30
_40_	4.59 × 10^11^	5.54 × 10^11^	9.26 × 10^10^	2.27
_54_	4.57 × 10^11^	3.83 × 10^11^	3.18 × 10^10^	2.35
_108_	5.22 × 10^11^	2.57 × 10^12^	1.36 × 10^11^	2.19
